# Are We There Yet? The Value of Deep Learning in a Multicenter Setting for Response Prediction of Locally Advanced Rectal Cancer to Neoadjuvant Chemoradiotherapy

**DOI:** 10.3390/diagnostics12071601

**Published:** 2022-06-30

**Authors:** Barbara D. Wichtmann, Steffen Albert, Wenzhao Zhao, Angelika Maurer, Claus Rödel, Ralf-Dieter Hofheinz, Jürgen Hesser, Frank G. Zöllner, Ulrike I. Attenberger

**Affiliations:** 1 Department of Diagnostic and Interventional Radiology, University Hospital Bonn, 53127 Bonn, Germany; ulrike.attenberger@ukbonn.de; 2 Computer Assisted Clinical Medicine, Mannheim Institute for Intelligent Systems in Medicine, Medical Faculty Mannheim, Heidelberg University, 68167 Mannheim, Germany; steffen.albert@medma.uni-heidelberg.de (S.A.); frank.zoellner@medma.uni-heidelberg.de (F.G.Z.); 3 Data Analysis and Modeling, Mannheim Institute for Intelligent Systems in Medicine (MIISM), Medical School Mannheim, Central Institute for Scientific Computing (IWR), Central Institute for Computer Engineering (ZITI), CZS Heidelberg Center for Model-Based AI, Heidelberg University, 69047 Heidelberg, Germany; wenzhao.zhao@medma.uni-heidelberg.de (W.Z.); juergen.hesser@medma.uni-heidelberg.de (J.H.); 4 Clinical Functional Imaging, Department of Diagnostic and Interventional Radiology, University Hospital Bonn, 53127 Bonn, Germany; angelika.maurer@ukbonn.de; 5 Department of Radiotherapy and Oncology, University Hospital Frankfurt, 60596 Frankfurt am Main, Germany; clausmichael.roedel@kgu.de; 6 Department of Medicine III, Medical Faculty Mannheim, Heidelberg University, 68167 Mannheim, Germany; ralf-dieter.hofheinz@medma.uni-heidelberg.de

**Keywords:** deep learning, machine learning, multicenter, locally advanced rectal cancer, response prediction to nCRT

## Abstract

This retrospective study aims to evaluate the generalizability of a promising state-of-the-art multitask deep learning (DL) model for predicting the response of locally advanced rectal cancer (LARC) to neoadjuvant chemoradiotherapy (nCRT) using a multicenter dataset. To this end, we retrained and validated a Siamese network with two U-Nets joined at multiple layers using pre- and post-therapeutic T2-weighted (T2w), diffusion-weighted (DW) images and apparent diffusion coefficient (ADC) maps of 83 LARC patients acquired under study conditions at four different medical centers. To assess the predictive performance of the model, the trained network was then applied to an external clinical routine dataset of 46 LARC patients imaged without study conditions. The training and test datasets differed significantly in terms of their composition, e.g., T-/N-staging, the time interval between initial staging/nCRT/re-staging and surgery, as well as with respect to acquisition parameters, such as resolution, echo/repetition time, flip angle and field strength. We found that even after dedicated data pre-processing, the predictive performance dropped significantly in this multicenter setting compared to a previously published single- or two-center setting. Testing the network on the external clinical routine dataset yielded an area under the receiver operating characteristic curve of 0.54 (95% confidence interval [CI]: 0.41, 0.65), when using only pre- and post-therapeutic T2w images as input, and 0.60 (95% CI: 0.48, 0.71), when using the combination of pre- and post-therapeutic T2w, DW images, and ADC maps as input. Our study highlights the importance of data quality and harmonization in clinical trials using machine learning. Only in a joint, cross-center effort, involving a multidisciplinary team can we generate large enough curated and annotated datasets and develop the necessary pre-processing pipelines for data harmonization to successfully apply DL models clinically.

## 1. Introduction

Colorectal cancer is the third most common cancer, accounting for 10% of all diagnosed cancers, and is the second leading cause of cancer death worldwide [1]. Rectal cancer constitutes about one-third of colorectal malignancies [2,3]. Within the last two decades, optimized imaging has enabled significant advances toward stage-specific and individualized therapy options for patients with rectal cancer.

The current standard-of-care treatment for locally advanced rectal cancer (LARC) involves neoadjuvant radiotherapy or chemoradiotherapy (nCRT) followed by total mesorectal excision (TME) [4]. Beyond reducing the local recurrence rate, improving resectability, and potentially preserving sphincter function in tumors of the lower rectum third, nCRT harbors the potential for an organ-preserving approach in patients exhibiting a clinical complete remission (cCR) [5]. Using standard chemoradiotherapy in locally advanced tumors, about 10% of patients demonstrate cCR [6]. Total neoadjuvant therapy (TNT), comprising 3–4.5 months of additional chemotherapy with 5-fluorouraicl and oxaliplatin before or after radio(chemo)therapy, increases the rate of cCR to about 30% [7]. Besides identifying patients who qualify for a watch-and-wait strategy, the reliable detection of treatment response is valuable for assessing overall survival [8,9].

Accurate staging/restaging using magnetic resonance imaging (MRI) to identify patients with a pathological complete response (pCR) prior to surgical resection is essential. cCR is used as a surrogate for pCR. However, treatment response assessment after nCRT by MRI is challenging and often entails overstaging due to mixed signal intensities or irregular fibrosis on T2-weighted (T2w) images and false positive residual lymph nodes [10]. The MRI-derived tumor regression grade (TRG) exhibits high specificity (93%) for pCR, but suboptimal sensitivity (32%) [11]. 

Considering these limitations of morphological MRI in evaluating treatment response, several studies have since investigated the potential of functional MRI like perfusion imaging [12,13] and, in particular, diffusion-weighted imaging (DWI) [14,15]. While DWI has been shown to increase diagnostic accuracy in detecting lymph node metastases and circumferential resection margin (CRM) involvement, its value in predicting treatment response is controversial [16,17,18]. Several models have been devised to quantitatively correlate the DWI signal with microstructural features of healthy and cancerous tissue in the rectum. 

The current clinical standard is the so-called apparent diffusion coefficient (ADC), which simply describes the DWI signal as decaying monoexponentially with increasing diffusion weighting [19]. Low pretherapeutic ADC values and an early increase in mean tumor ADC values during therapy were found to correlate with a good treatment response [17,20,21,22,23]. However, other studies failed to reproduce this correlation or even demonstrated an inverse correlation linking pretherapeutic high ADC values to a good response to nCRT and initial low ADC values to increased tumor aggressiveness [14,17,24,25,26,27,28,29,30]. Focal diffusion restriction at DWI may be observed in up to 50% of patients at the site of proven pCR [10]. Furthermore, there is a high overlap of ADC values in patients with pCR and partial regression [31].

With the increasing availability of graphical processing units, the application of Deep Learning (DL) to train neural networks holds a very promising, now feasible approach to improve the hitherto insufficient characterization of therapy response relying on T2w and DWI [6]. In a single-center study with external validation, Jin et al. designed two Siamese subnetworks joined at multiple layers to allow for simultaneous tumor segmentation and response prediction [32]. After training their model using pre- and post-therapeutic T2w, diffusion-weighted (DW) and T1w with and without contrast enhancement images of 321 Asian LARC patients from one center, they prospectively obtained a dataset of 160 Asian LARC patients treated at the same center for internal validation. In an independent, external validation cohort of 141 Asian LARC patients from another institution, they achieved high accuracy in predicting pCR with an area under the receiver operating characteristic curve (AUC) of 0.92 (95% confidence interval (CI): 0.87–0.96). Integrating blood-based tumor markers further improved prediction accuracy to an AUC of 0.97 (95% CI: 0.93–0.99).

Before translating such DL approaches into clinical routine for screening, treatment response assessment and surveillance, their predictive performance remains to be verified in a multicenter setting with heterogeneous data of different ethnic groups. Facing the challenge of varying data quality and uniformity, in this study we test a DL network similar to the approach by Jin et al. [32] on a multicenter dataset from a Western population. To ensure clinical feasibility, we train and validate our network using only pre- and post-therapeutic T2w images, DW images at b = 800 s/mm^2^ and ADC-maps of 83 LARC patients from four different centers and test our trained network on an independent, external, clinical routine dataset of 46 LARC patients. In this work, we aim to share our initial experiences and, more importantly, challenges of applying a state-of-the-art DL model to a multicenter dataset. Only through international collaborations and joint efforts will we achieve broad applicability of DL that will have a clinical impact.

## 2. Materials and Methods

### 2.1. Patient Cohort

**Figure 1:**
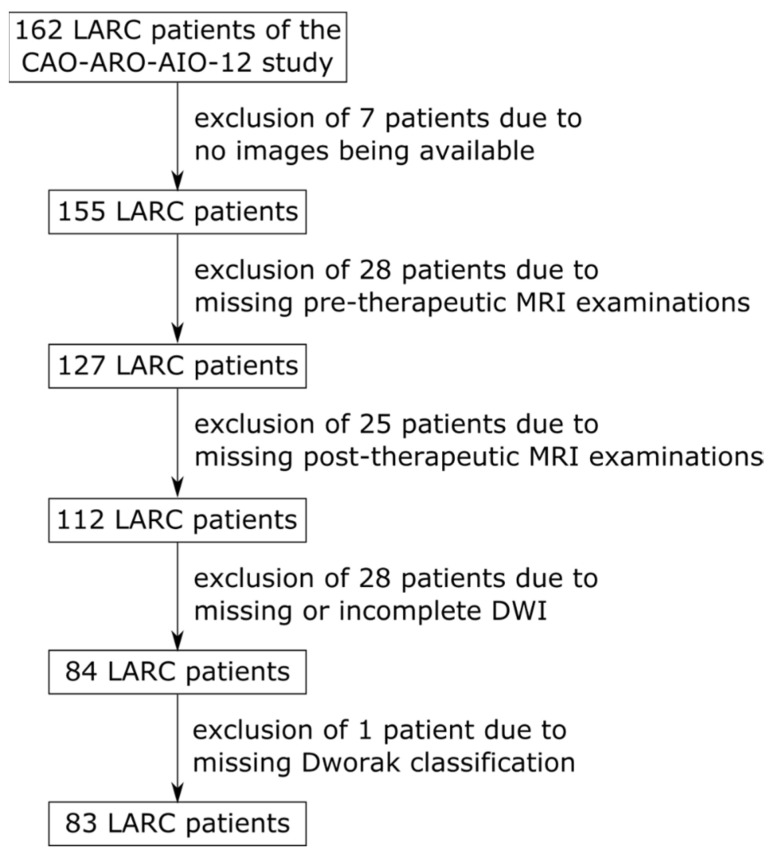
Flowchart of the inclusions and exclusions of LARC patients of the CAO-ARO-AIO-12 study.

With the approval of the institutional review board, this retrospective study enrolled 162 LARC patients (59 female mean age 63 years +/− 11 years 2 standard deviations, 103 male mean age 60 years +/− 10 years 2 standard deviations) of the CAO-ARO-AIO-12 study [33,34] acquired between the years 2015 and 2018 (Figure 1). The following centers participated in this study: Center 1 (Frankfurt), Center 2 (Regensburg), Center 3 (Würzburg), Center 4a (Mannheim). Furthermore, a clinical routine dataset acquired at Center 4b (Mannheim) from 2009 to 2013 without study conditions of 46 LARC patients was retrospectively included as an external test dataset in our DL approach.

The inclusion criteria of this study were: (a) histopathologically proven LARC, (b) treatment with nCRT followed by TME and pathological assessment of the TRG, (c) availability of pre- and post-nCRT MRI examinations including axial, high-resolution T2w- and DWI-sequences with the calculation of ADC maps. Patients had to be excluded if their MRI dataset was either incomplete or the pathologic TRG was not available.

### 2.2. Image Acquisition and Radiologic Assessment

All patients underwent a clinically indicated MRI examination before and after nCRT. As per the study protocol, MR images were acquired with at least a 1.5 T, preferably a 3.0 T MRI scanner in the supine position and with a phased-array body coil wrapped on the surface of the patient to cover the entire pelvis. Suggested parameters for the MR sequences relevant to this study were:

Axial T2w-TSE: Repetition time (TR) 5600 ms, echo time (TE) 110 ms, max. 3 mm slice thickness, voxel size 0.8 × 0.8 × 3 mm.

Axial DWI-EPI: TR 6500 ms, TE 76 ms, max. 5 mm slice thickness, voxel size 1.8 × 1.8 × 4 mm, acquired b-values 50/400/800 s/mm^2^ and a calculated b-value of 1400 s/mm^2^.

All MRI examinations before and after nCRT were clinically assessed and evaluated by trained, board-certified radiologists at the respective centers prior to surgery, avoiding knowledge of histopathology.

The tumor region on pre- and post-therapeutic, axial, high-resolution T2w images was segmented by a trained radiologist with 3 years of experience using the freely available, open-source software ITK-SNAP [35,36].

### 2.3. Pathological Assessment of Tumor Regression

After TME, the surgically resected tumor specimens were histopathologically examined and pathologic TRG was determined according to the Dworak classification [37]. The grading system was established as follows: TRG 0—no regression; TRG 1—dominant tumor mass with obvious fibrosis and/or vasculopathy; TRG 2—dominantly fibrotic changes with few tumor cells or groups (easy to find); TRG 3—very few (difficult to find microscopically) tumor cells in fibrotic tissue with or without mucous substance; TRG 4—no tumor cells, only fibrotic mass (total regression or response) [37].

### 2.4. Data Processing

Three image processing and harmonization steps were applied toward enhancing the robustness of the model. To correct for B1 field inhomogeneities, bias correction was performed using the N4 bias correction [38] implemented in SimpleITK [39,40]. Subsequently, rigid image registration of pre- and post-therapeutic T2w images was performed using Advanced Neuroimaging Tools (ANTs) [41] with standard parameters and without a mask. Their exact transformation was then applied to the DW images with SimpleITK. Alignment between T2w and DW images was visually assessed. Center-cropping and normalization was performed using the code provided by Jin et al. [32].

### 2.5. Deep Learning Model

For the classification task at hand, we used the approach developed by Jin et al. [32]. The code corresponding to the paper was taken from the repository and only the number of input channels was changed to match our training data of pre- and post-therapeutic T2w and DW images. The model was trained in two configurations: (1) using only T2w images as input, (2) using T2w and DW images comprising a b800 DW image and an ADC map as input. 

The model utilizes multitask learning to improve its performance. Two U-Nets with shared parameters are used to segment the tumor in the pre- and post-therapeutic images. Features are then extracted from both networks at multiple levels. To evaluate treatment response, differences between these features are used for classification after applying a depth-wise convolution. Patients were classified into two classes: pCR (TRG 4) and non-pCR (TRG 0–3).

### 2.6. Training

**Figure 2:**
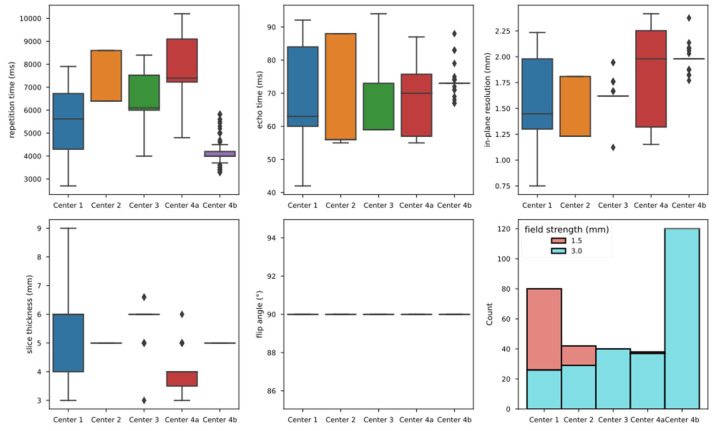
Distribution of selected imaging parameters per center for the T2w images.

The model was trained on all patients of Centers 1, 2, and 3 as described by Jin et al. [32], i.e., for 1000 epochs using focal loss and an initial learning rate of 0.01, which was halved when the loss did not improve for 30 epochs. To prevent overfitting, the weights of the epoch with the highest AUC on the validation set were used. An exponential moving average was applied to the validation score to remove random fluctuations. Due to the small size of the dataset, the training cohort was evaluated using 5-fold cross-validation. For each fold, the data of Centers 1, 2, and 3 were split into 68% for training, 12% for validation and 20% for testing, resulting in five trained networks. To test the performance of our approach, the data of Center 4a, which were previously unseen by the network, were applied to each of the previously trained networks for prediction. To evaluate model generalizability, the network was tested on a clinical routine dataset of 46 LARC patients from Center 4b, which were outside the training distribution (Figure 2). Again, these data were applied on the five previously trained networks for prediction. 

### 2.7. Statistical Analysis

The confidence interval for the AUC of each fold was calculated using bootstrapping with 1000 samples. Random samples (allowing for duplicates), with the same number of patients as the original set, were drawn from the scores predicted by the network. Samples that did not include patients with pCR were skipped. The results were used for error calculation of the AUC.

The fast implementation of De Long’s method was used to calculate if there were significant differences in the AUC between the models [42]. The significance of the differences in patients’ characteristics was calculated using the Mann–Whitney U-Test with the null hypothesis that the distributions are equal. A *p*-value of 0.05 was considered significant.

## 3. Results

### 3.1. Patients’ Characteristics

There were significant differences in the distribution of patients’ characteristics, including age, pre- and post-nCRT MRI staging, tumor localization and time interval between initial MRI staging, re-staging and the date of the operation. For a comprehensive overview of patients’ characteristics in the training, validation and test cohorts, please refer to Table 1.

### 3.2. Imaging Characteristics

Apart from the clinical routine test dataset, various scanners of different manufacturers with dissimilar field strengths were used at each center, and in many cases the same patient was examined on differing scanners before and after nCRT. In addition, over the course of the CAO-ARO-AIO-12 study, selected scanners were upgraded, e.g., the Avanto scanner at Center 1 was upgraded to the Avanto-fit scanner. Assuming that each center used only one scanner with the same model name, 15 different scanners were used at 4 centers (see Table 2).

Furthermore, we observed significant differences in imaging parameters between the training/validation cohort and the test cohort, some of which also differed significantly from the prespecified study protocol (Table 3). Figure 2 exemplifies the distribution of selected parameters in T2w imaging across the different centers. It is of note that even within a single-center, parameters sometimes varied significantly. Consequently, the imaging data of the included patients from the CAO-ARO-AIO-12 study were very heterogeneous in terms of image quality.

### 3.3. Data Processing

Rigid registration between the T2w pre- and post-therapeutic images was overall successful by visual inspection. However, we noticed a mismatch of the DW to the T2w images despite them being acquired in the same session. Upon closer examination, we found that, in addition to motion artifacts, misaligned slice positioning and angulation between the T2w and DW images played a major role in this. Attempts to correct this misalignment by rigid registration failed, presumably due to low SNR and distortions afflicting all DW images probably caused by susceptibilities affecting the EPI acquisition.

### 3.4. Model Performance

**Figure 3:**
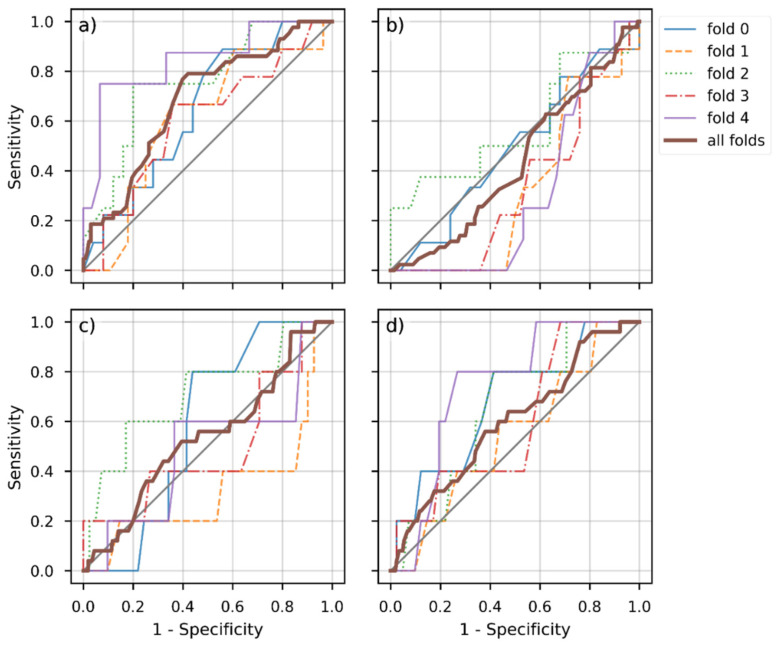
Receiver operator characteristic (ROC) of all obtained models (i.e., folds) and combination of input data for the training (**a**,**b**) and external validation dataset (**c**,**d**). (**a**,**b**) depict ROC curves for the training cohort: (**a**) using only T2w images as input vs. (**b**) using T2w and DW images as input. (**c**,**d**) depict the corresponding results for the external validation dataset: (**c**) using only T2w images as input vs. (**d**) using T2w and DW images as input. In each panel, the bold brown-colored curve depicts the average over the five folds.

The AUC for pCR prediction averaged over the five models obtained via cross validation using only pre- and post-therapeutic T2w images as input was 0.68 (95% confidence interval [CI]: 0.59, 0.77). Combining pre- and post-therapeutic T2w, DW images, and ADC maps as input yielded an AUC of 0.43 (95% CI: 0.35, 0.52). Figure 3a and c depict the receiver operator characteristic (ROC) curves for each of the five models and the average over all folds.

Testing our implementation of the network of Jin et al. [32] on the external dataset (Center 4b) returned an AUC for pCR prediction of 0.54 (95% CI: 0.41, 0.65), when using only pre- and post-therapeutic T2w images as input. Using the combination of pre- and post-therapeutic T2w, DW images, and ADC maps as input, the AUC for pCR prediction was 0.60 (95% CI: 0.48, 0.71). Figure 3b and d depict the ROC curves for each of the five models and the average over all folds.

**Figure 4:**
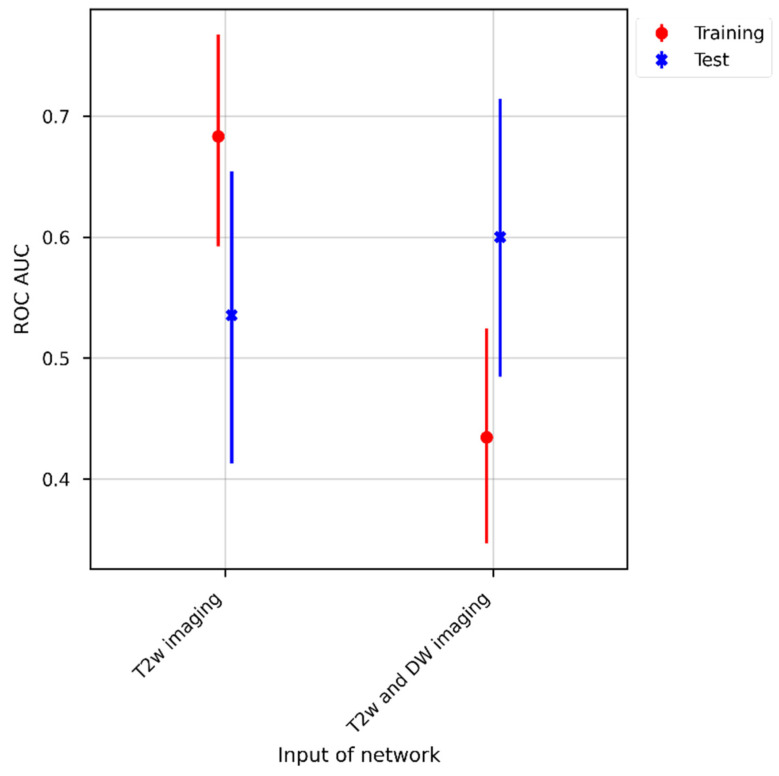
The area under the receiver operating characteristic curve (AUC) was calculated for the different receiver operating characteristic curves depicted in Figure 3. The AUCs are shown for the training (colored red) and test cohorts (colored blue). For the training cohort, classification with only T2w images as inputs performed significantly better than with T2w and DW images.

For the different ROC curves depicted in Figure 3, the AUC was calculated and plotted in Figure 4. In the training cohort, classification using only the T2w images performed significantly better than using both T2w and DW images (*p* < 0.01 using the DeLong method). In the external dataset, using both T2w and DW images yielded the best results with an AUC of 0.68, but was not significantly better than using only T2w images (*p* = 0.3 using the DeLong method).

**Figure 5:**
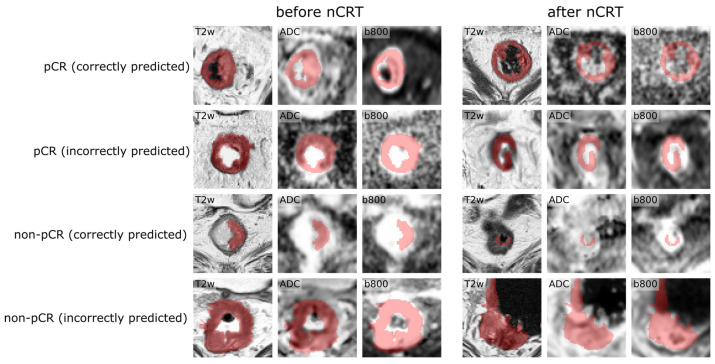
Examples of correctly and incorrectly classified patients. Each row shows input images of one patient before and after nCRT overlayed with the segmentation mask. Note the slight misalignment of the segmentation mask with the tumor region on the DW images, which might contribute to misclassification.

To review model performance, we overlaid the different input images with the segmentation mask originally annotated on the high-resolution T2w images (Figure 5). As described above, the morphological images and the DW images were at times misaligned, so that the transformed segmentations incompletely or incorrectly labeled the tumor region on the DW images. In these cases of misalignment, the performance of the algorithm dropped considerably as illustrated by Figure 5.

## 4. Discussion

Reliably predicting treatment response/pCR preoperatively after completion of nCRT using MRI holds the potential to offer LARC patients a watch-and-wait strategy instead of surgical resection and to better assess overall survival. DL provides the ability to train a neural network to identify patients with pCR using morphological and functional imaging data as input [6]. While initial results from single-center studies are promising [32,43], further studies using multicenter real-world data are necessary before such networks can be widely applied clinically. In this study, we retrained a promising, state-of-the-art multitask DL model [32] using a heterogenous dataset consisting of T2w, DW images and ADC maps acquired before and after nCRT and assessed its performance in a multicenter trial. 

Our results show that the performance of the proposed DL model decreases significantly using a multicenter dataset. Three key takeaways can be concluded from our study:

1. Data quality and uniformity are pivotal features to be addressed in clinical trials involving machine learning, requiring the development of a dedicated pre-processing pipeline.
2. If no homogeneous data are available, the sample size for training the DL approach needs to be drastically increased to mitigate artifacts related to image inhomogeneity.
3. Translating DL models into potentially useful clinical tools requires cross-center involvement of a multidisciplinary team.

We aimed to answer the question to what extent translation of promising DL models into the clinic is possible. Thus, to ensure clinical feasibility, we decided to use only pre- and post-therapeutic T2w and DW images as inputs for the proposed DL model and to refrain from collecting additional parameters such as blood values. Despite these minimal requirements, 78 of 162 patients of the CAO-ARO-AIO-12 study dataset already had to be excluded due to missing or incomplete image datasets that had been provided to us. 

To train the network, we used patients enrolled under study conditions from the CAO-ARO-AIO-12 study [33], which has certain standards for image acquisition. Yet, significant differences in the composition of the patient population as well as in the distribution of the acquisition parameters were evident in these data. These differences were even more pronounced when comparing the dataset acquired under study conditions with the external, clinical dataset that we used for testing. Differences in image acquisition parameters result in varying image quality. The problem of data heterogeneity is compounded by the fact that the image datasets in this study were acquired on a total of 15 different scanner types of two vendors with varying field strengths. Often an individual patient was even examined on different scanners before and after nCRT. Particularly, the DW images often exhibited poor and heterogeneous quality, and moreover, were often acquired in a dissimilar orientation relative to the T2w images. These factors, including a low signal-to-noise ratio, various artifacts, and low resolution, led to the fact that we failed to register the DW images sufficiently with the high-resolution T2w images [44]. This proved especially challenging as tumor segmentation was performed on the high-resolution T2w images. Notably, for those patients misclassified by the network, the correspondence of the segmentation to the tumor region in the DW images was poor. It should also be pointed out that, especially in the internal multicenter training dataset, the performance of the network decreased from an AUC of 0.68 to 0.43 when the DWI data were added as an input to the pre- and post-therapeutic T2w images. Interestingly, this was not the case in the single-center test dataset. 

These observations are consistent with the findings of Schurink et al. [45], who found significant intercenter variation in multicenter rectal MRI data, with greater variation in DW images and ADC maps compared to T2w images, mainly related to hardware and image acquisition protocols. They further state that such variations will likely negatively influence subsequent analysis if not corrected for [45]. Even though imaging processing and data harmonization were applied in this study prior to network training and testing, our results indicate that further development of a dedicated data pre-processing pipeline is necessary that specifically addresses the inhomogeneities of a multicenter dataset [46,47]. As an analysis by Glocker et al. suggests, current approaches to harmonizing data are not able to correct for scanner-specific biases and harmonization of data remains an open challenge [48]. 

Furthermore, to mitigate artifacts related to image inhomogeneity, much larger datasets are required for network training [49,50,51]. Yet, the availability of image data seems to be one of the biggest hurdles for the implementation of artificial intelligence in the clinical setting [52], as supported by our study. In addition, data preparation and annotation are very costly and time consuming [52]. Small datasets from small geographic areas are prone to selection bias that can lead to algorithms with limited utility and poor generalizability [52]. While new approaches such as federated learning might be able to address the problem of data availability, data curation and annotation accounted for a significant portion of our work, which pose barriers to a successful implementation of such algorithms in clinical practice [52]. In this regard, we consider it very important to emphasize that, beyond the arguments already mentioned, the basis or prerequisite for a successful translation of AI algorithms into the clinic is a cross-center, interdisciplinary team. This begins with careful planning by a multidisciplinary team and the specification of acquisition parameters that are implemented by radiographers, continues with standardized reporting by radiologists, cross-center image processing and preparation by data scientists, and finally ends with interdisciplinary interpretation of the results. Only through a joint effort will a sustainable implementation of such promising DL models in the clinic succeed.

Our study has certain limitations. Image registration of the image data turned out to be challenging due to distortions and slice misalignment within a patient rather than between time points. So far, we only used a rigid registration to align pre- and post-therapeutic images similar to Jin et al. [32]. In their work, it seems that the issue of distortions and misalignment between T2w and DWI was either not dealt with or minor. Due to the small size of our dataset and its heterogeneity, an elastic image registration would probably enhance our classification results which will be future research. 

In comparison to the study by Jin et al. [32], we reduced the number of network inputs, partly because the CAO-ARO-AIO-12 study dataset, for instance, did not include pre- and post-contrast T1w images for all patients, and partly to ensure clinical feasibility, as already written above. Furthermore, we were limited in our ability to test the generalizability of the DL model [32] because the weights of the pre-trained network were not shared with us. This is an obstacle in the applicability of many published networks.

Lastly, we did not optimize the hyperparameters of the network, which might boost the performance since we aimed at translating the model “as is” to our local settings in the best possible way.

## 5. Conclusions

In this study, we re-trained a promising state-of-the-art multitask DL model using a heterogeneous multicenter dataset and assessed its performance in predicting pCR of LARC to nCRT using an external, real-world clinical dataset. Our results demonstrate that despite dedicated data pre-processing, the performance of the DL network drops significantly in the multicenter setting compared to the original single-center study with external validation. This finding highlights the importance of data quality and harmonization in clinical trials using machine learning. Only in a joint, cross-center effort, involving a multidisciplinary team can we generate large enough curated and annotated datasets and develop the necessary pre-processing pipelines for data harmonization to successfully apply DL models clinically. 

## Figures and Tables

**Table 1:** Characteristics of patients in the training, validation and test cohort disaggregated by centers.

	Training Cohort			Validation Cohort	Test Cohort	Significant Differences
Characteristic	Center 1	Center 2	Center 3	Center 4a	Center 4b	
Acquisition (years)	2015–2018	2016–2017	2015–2017	2015–2017	2009–2013	**0.00**
Age (mean ± std)	60 ± 10	61 ± 11	60 ± 6	66 ± 6	64 ± 11	0.07
**Sex**						0.15
Male	26	14	8	9	37	
Female	11	6	7	2	9	
**Pre-nCRT T-stage (MRI)**						**0.02**
T0	0	0	0	0	0	
T1	0	0	0	0	0	
T2	3	0	2	0	8	
T3	28	1	11	5	38	
T4	4	0	1	0	0	
Not specified	2	19	1	6	0	
**Pre-nCRT N-stage (MRI)**						**0.00**
N-	2	2	0	1	25	
N+	35	18	15	10	21	
**CRM (initial staging)**						0.06
Minimal distance to mesorectal fascia (MRF) in mm	12 ± 9		2 ± 5		3 ± 3	
MRF involvement	2	0	0	2	7	
Not specified	13	20	0	9	0	
**Tumor location**						**0.0**
lower third	18	5	5	4	10	
middle third	13	9	10	5	25	
upper third	0	0	0	0	11	
location not specified	6	6	0	2	0	
**Post-nCRT T-stage (MRI)**						**0.03**
T0	1	0	3	0	0	
T1	2	0	1	0	2	
T2	13	0	1	0	27	
T3	17	0	10	0	17	
T4	4	0	0	1	0	
Not specified	0	20	0	10	0	
**Post-nCRT N-stage (MRI)**						0.68
N−	17	0	2	0	39	
N+	20	0	13	2	5	
Not specified	0	20	0	9	2	
**pCR**						0.15
pCR	7	9	1	2	5	
Non-pCR	30	11	14	9	41	
**Time in days (mean ± std)**						
Initial Staging to OP	146 ± 12	146 ± 11	142 ± 8	177 ± 35	123 ± 20	**0.0**
Post-nCRT MRI to OP	13 ± 10	7 ± 3	8 ± 3	32 ± 15	29 ± 14	**0.0**

Unless otherwise indicated, data are number of patients. Significance of differences between the training/validation and test cohort were calculated using the Mann–Whitney U-test; a *p*-value below 0.05 was considered significant.

**Table 2:** Overview of the different scanners used for imaging the patients at each center.

	Vendor	Model Name	Tesla	Number of Patients before nCRT	Number of Patients after nCRT
**Center 1**	Siemens	Prisma_fit	3.0	6	18
	Siemens	Avanto	1.5	14	0
	Siemens	Avanto_fit	1.5	2	14
	Siemens	SymphonyTim	1.5	12	1
	Siemens	Aera	1.5	1	3
	Siemens	Espree	1.5	0	1
	Philips	Ingenia	1.5	1	0
	Siemens	Spectra	3.0	1	0
**Center 2**	Siemens	Skyra	3.0	17	10
	Siemens	Avanto	1.5	3	10
**Center 3**	Siemens	Prisma_fit	3.0	11	9
	Siemens	Skyra	3.0	4	6
**Center 4a**	Siemens	Skyra	3.0	4	8
	Siemens	TrioTim	3.0	6	3
	Siemens	Avanto	1.5	1	0
**Center 4b**	Siemens	TrioTim	1.5	46	46

**Table 3:** Differences between the training and test dataset.

	Training T2w	Test T2w	Training DWI	Test DWI
**Slice thickness (mm)**	3.3 ± 0.5	3.1 ± 0.2	5.1 ± 1.1	5.0 ± 0.0
**Repetition time (ms)**	4994.9 ± 1913.5	3971.5 ± 708.1	6463.1 ± 1539.1	4121.9 ± 562.7
**Pixel bandwidth (Hz)**	215.9 ± 48.9	201.6 ± 8.8	1792.5 ± 351.6	1735.0 ± 8.3
**Flip angle (°)**	137.0 ± 16.5	148.7 ± 5.6	90.0 ± 0.0	90.0 ± 0.0
**Echo time (ms)**	97.8 ± 14.5	101.9 ± 4.7	67.2 ± 13.4	73.2 ± 2.2
**Field strength (T)**	2.5 ± 0.7	3.0 ± 0.0	2.5 ± 0.7	3.0 ± 0.0
**In-plane resolution (mm)**	0.6 ± 0.2	0.6 ± 0.0	1.6 ± 0.4	2.0 ± 0.1

Each column shows the mean and standard deviation of the different acquisition parameters of the T2w and DW sequences for the training and test cohort, respectively. Only the flip angle (90°) and pixel bandwidth of DWI were not significantly different between cohorts.

## Data Availability

Restrictions apply to the availability of these data. Data was obtained from German Rectal Cancer Study Group (EudraCT No.: 2011-006310-13). The data are not publicly available due to patient privacy.
